# Morphological and molecular characterization of *Xiphinemella esseri* Chitwood, 1957 (Dorylaimida: Leptonchidae) from Florida, with the first molecular study of the genus

**DOI:** 10.21307/jofnem-2021-032

**Published:** 2021-03-20

**Authors:** Sergio Álvarez-Ortega, Sergei A. Subbotin, Renato N. Inserra

**Affiliations:** 1 Departamento de Biología y Geología, Física y Química Inorgánica, Universidad Rey Juan Carlos, Campus de Móstoles, 28933, Madrid, Spain; 2 Plant Pest Diagnostic Centre, California Department of Food and Agriculture, 3294 Meadowview Road, Sacramento, CA, 95832-1448; 3 Centre of Parasitology of A.N Severtsov Institute of Ecology and Evolution of the Russian Academy of Sciences, Leninskiy Prospect 33, Moscow, 117071, Russia; 4 Florida Department of Agriculture and Consumer Services, DPI, Nematology Section, P.O. Box 147100, Gainesville, FL, 32614-7100

**Keywords:** 18S rRNA, 28S rRNA, Bayesian inference, Description, D2-D3 expansion segments, Molecular, Morphology, Morphometrics, Taxonomy

## Abstract

A population of *Xiphinemella esseri*, recently collected under the canopy of associated live oak trees in north Florida, was studied and described with an integrative approach, including the first molecular study of the genus. This Florida population is characterized by its 2.30 to 3.32 mm long body, labial disc well developed, lip region offset by constriction, and 16.5 to 17.5 μm broad, odontostyle 46 to 49 μm long with minute aperture, neck 288 to 296 μm long, pharyngeal expansion occupying 28 to 30% of total neck length, uterus a tripartite tube-like structure, *pars refringens vaginae* absent, vulva transverse (*V* = 45.4-49.7%), tail short and rounded (18-28 μm, *c* = 94-158, *c′* = 0.6-0.9), spicules 41 to 45 μm long, and 8 to 10 irregularly spaced ventromedian supplements bearing hiatus. The phylogenetic analysis inferred from the D2-D3 expansion segments of 28S rRNA gene and 18S rRNA gene sequences showed that *X. esseri* clustered with other dorylaims from the family Leptonchidae. A brief discussion about the distribution and biological considerations of *X. esseri* is also provided.

The genus *Xiphinemella* (Loos, 1950) is a rather rare dorylaimid taxon, which has been reported in Africa, Asia, Europe, and North America (Andrássy, 2009). Species of this genus have cuticle consisting of an irregularly outlined inner layer with refractive elements, and smooth outer layer as in others tylencholaimoids, lip region with a distinct labial disc, odontostyle long and attenuated, and odontophore with prominent basal flanges. In total, 11 valid species were listed by De Bruin and Heyns (1991) in their thorough review of *Xiphinemella.* Three additional species were added later by Choi et al. (1992), Dhanachand et al. (1991), and Loof and Zullini (2000). Peña-Santiago (2006) regarded the genus *Zalophidera* (Siddiqi, 1982) a junior synonym of *Xiphinemella* and transferred the two valid species of *Zalophidera* into *Xiphinemella* increasing to 16 the number of valid species in this genus. *Xiphinemella* is currently placed under the subfamily Xiphinemellinae (Jairajpuri, 1964), one of the four subfamilies classified under the family Leptonchidae (Thorne, 1935), but its relationships with the others leptonchid genera have not been analyzed using molecular data yet, due to lack of DNA sequences of the Xiphinemellinae members. This study was conducted with the objectives to (i) characterize a population of *Xiphinemella esseri* (Chitwood, 1957), recently collected in Florida, with molecular and morphological taxonomic approach, and (ii) provide new insights in the phylogeny and taxonomy of the genus *Xiphinemella*.

## Materials and methods

### Sampling, extraction, and morphological identification

Nematode populations used in this study were obtained from four samples collected under the canopy of associated live oak (*Quercus virginiana* Mill.) trees in north Florida. Nematodes were extracted from soil using the centrifugal flotation method (Jenkins, 1964). All the extracted needle nematodes were used for morphological and molecular analyses. Nematodes were killed and fixed using the Golden’s method described in Southey (1970). Live specimens were hand-picked with an eye lash and transferred into 2 to 3 ml of distilled water in a watch glass and put in an oven at 43°C. After 15 min the watch glass was filled with fixative (a water solution of 3% formaldehyde and 2% glycerol) that was kept in the oven at the same temperature. The watch glass was then enclosed in a petri dish and kept in oven to allow a slow evaporation of the fixative and the infiltration of the nematode with glycerol for three to five days. Specimens were then mounted on permanent glass slides to allow handling and observation under LM.

Measurements of specimens were taken with an Olympus BX51 (Olympus, Tokyo, Japan) ocular micrometer. Morphometrics included de Man’s indices and standard measurements. Spicule terminology used follows Peña-Santiago et al. (2014). Some of the best-preserved specimens were photographed with the same microscope equipped with differential interference contrast and a Canon EOS 250D digital camera (Canon, Tokyo, Japan). Digital images were edited using Adobe^®^ Photoshop^®^ CS (Adobe Systems, San Jose, CA).

### DNA extraction, PCR, sequencing, and phylogenetic analysis

DNA was extracted from single individuals using the proteinase K protocol. DNA extraction, PCR, and cloning protocols were used as described by Tanha Maafi et al. (2003). The primers used for amplification of the D2-D3 expansion segments of 28S rRNA gene were the D2A (5′-ACA AGT ACC GTG AGG GAA AGT TG-3′) and the D3B (5′-TCG GAA GGA ACC AGC TAC TA-3′) (Subbotin et al., 2006). The 18S rRNA gene was amplified using two sets of primers (two overlapping fragments): (i) forward G18SU (5′-GCT TGT CTC AAA GAT TAA GCC-3′) and reverse R18Tyl1 (5′-GGT CCA AGA ATT TCA CCT CTC-3′) and (ii) forward F18Tyl2 (5′-CAG CCG CGG TAA TTC CAG C-3′) and reverse R18Tyl2 (5′-CGG TGT GTA CAA AGG GCA GG-3′)(Chizhov et al., 2006). PCR products were purified using the QIAquick PCR purification Kit (Qiagen) and used for direct sequencing. The sequencing was performed at GENEWIZ (San Francisco, CA, USA). The newly obtained sequences were submitted to the GenBank database under accession numbers: MW221076, MW221077, MW590305. The newly obtained sequences of the D2-D3 of 28S rRNA and 18S rRNA genes were aligned using ClustalX 1.83 (Thompson et al., 1997) (the D2-D3 of 28S rRNA alignment parameters: gap opening – 5, gap extension – 3; 18S rRNA alignment parameters: gap opening – 15, gap extension – 6.66) with published gene sequences (Holterman et al., 2008; Mullin, 2004; van Megen et al., 2009). All sequence alignments were analyzed with Bayesian inference (BI) using MrBayes 3.1.2 (Ronquist and Huelsenbeck, 2003) under the GTR + G + I model. BI analysis was initiated with a random starting tree and was run with four chains for 8.0 × 10^6^ generations for the D2-D3 of 28S rRNA alignment and 3.0 × 10^6^ generations for the 18S rRNA alignment. Posterior probabilities (PP) in percentage are given on appropriate clades.

## Results and description

### Systematics

***Xiphinemella esseri*** (Chitwood, 1957).

Figure 1.

**Figure 1:**
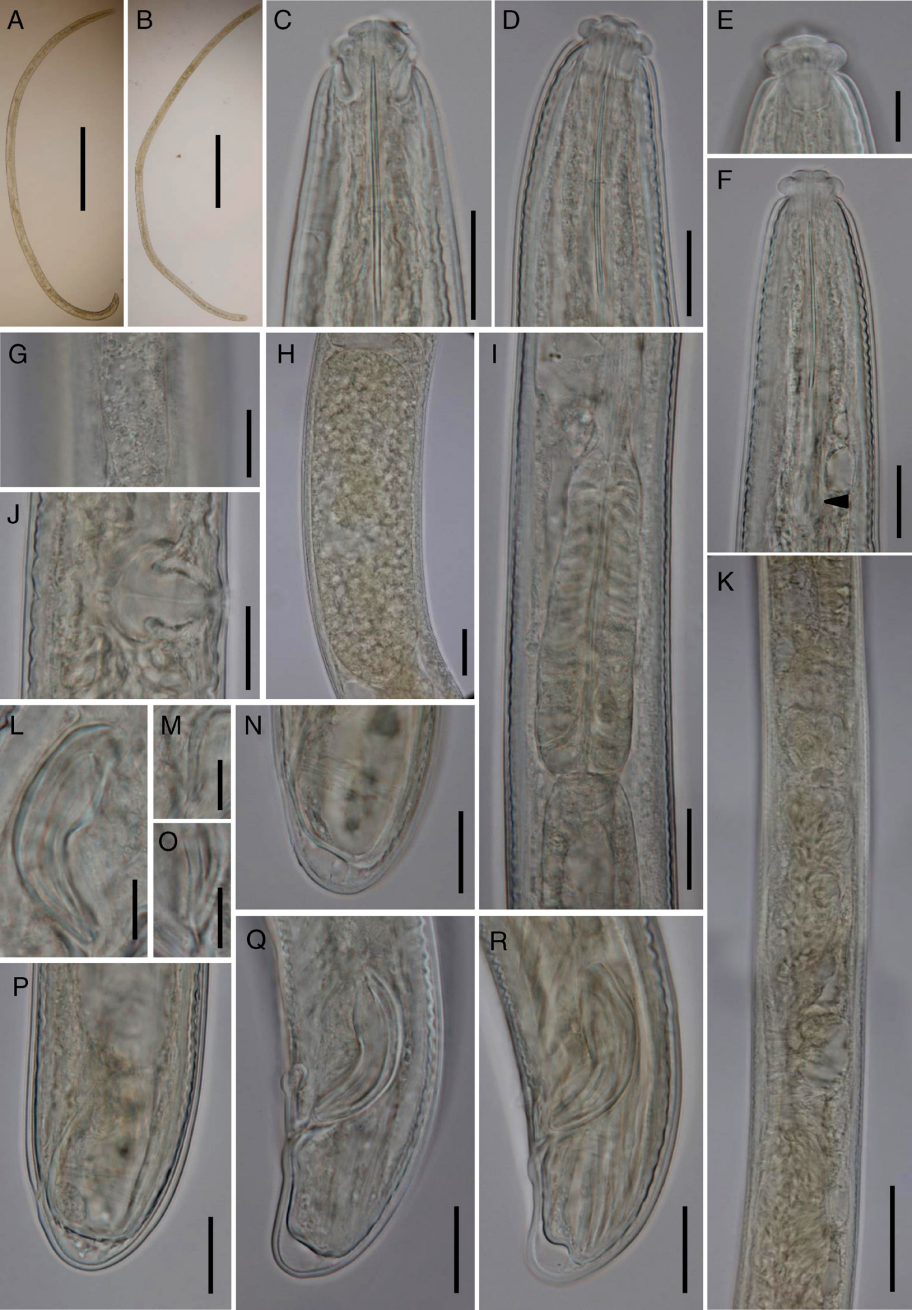
*Xiphinemella esseri* (Chitwood, 1957) (light photomicrographs). A: Male, entire body; B: Female, entire body; C: Anterior region in dorso-ventral median view; D, F: Anterior region in lateral median view (F, arrow pointing on odontophore basal flanges); E: Lip region in surface lateral view; G: Lateral chord; H: Uterine egg; I: Pharyngeal expansion; J: Vagina; K: Anterior genital branch in part (showing the oviduct-uterus junction and uterus morphology); L: Spicule; M, O: Lateral guiding piece; N, P: Female caudal region; Q, R: Male caudal region. (Scale bars: A, B = 500 μm; C, D, F, G, I-K, N, P-R = 20 μm; E, L, M, O = 10 μm; H = 25 μm).

### Material examined

Nine females and seven males in good state of preservation.

### Measurements

See Table 1.

**Table 1.** Morphometrics of *Xiphinemella esseri* Chitwood, 1957.

Population		Florida, USA					
Reference		Present paper		Chitwood (1957)		De Bruin and Heyns (1991)	
Character	*n*	9♀♀	7♂♂	♀♀	♂♂	5♀♀	4♂♂
*L*		2.82 ± 0.23 (2.47-3.32)	2.55 ± 0.26 (2.30-3.07)	2.4–3.5	2.2-3.0	3.10 (2.62-3.74)	3.17 (3.06-3.25)
*a*		53.2 ± 3.1 (50-59)	52.1 ± 6.4 (45-62)	40-43	35-45	47.1 (39.7-58.4)	53.2 (51.6-54.2)
*b*		8.6 (*n* = 1)	8.2 (*n* = 2)	11.4-14.0	7.6-11.0	10.3 (8.7-11.6)	10.8 (9.9-12.2)
*c*		117 ± 21 (100-158)	101 ± 5.5 (94-110)	93-130	77-120	137 (95-161)	124 (105-152)
*c′*		0.7 ± 0.1 (0.6-0.8)	0.8 ± 0.1 (0.7-0.9)	0.8^a^	0.9^a^	0.6 (0.5-0.7)	0.85 (0.7-1.0)
*V*		47.3 ± 1.5 (45.4-49.7)	–	42-47	–	46 (45-47)	–
Lip region diam.		16.8 ± 0.4 (16.5-17.5)	16.6 ± 0.2 (16.5-17.0)	17^a^	–	17.2 (17-18)	17 (16-18)
Odontostyle length		47.0 ± 1.0 (46-49)	46.3 ± 0.4 (46-47)	72-75^b^	–	46 (42-50)	46 (45-48)
Odontophore length		35.0 ± 0.8 (34-36)	34.9 ± 1.0 (33-36)	–	–	33 (31-35)	34 (31-36)
Guiding ring from ant. end		36.3 ± 1.5 (34-39)	36.5 ± 1.0 (35-38)	35-38	–	36 (30-42)	37 (32-42)
Neck length		288 (*n* = 1)	288, 296 (*n* = 2)	–	–	300 (276-333)	296 (267-322)
Pharyngeal expansion length		84.0 ± 2.8 (81-87)	82.2 ± 1.9 (80-85)	75-85	–	84 (80-89)	80 (76-85)
Diam. at neck base		45.1 ± 2.8 (42-50)	44.6 ± 2.5 (42-48)	–	–	–	–
at midbody		53.4 ± 4.8 (49-64)	50.0 ± 3.4 (46-55)	–	–	–	–
at anus		34.0 ± 2.6 (32-39)	30.4 ± 1.2 (29-32)	–	–	39 (30-42)	31 (30-32)
Prerectum length		611, 683 (*n* = 2)	594 ± 142,3 (444-727) (*n* = 3)	–	–	557 (427-695) (*n* = 3)	–
Rectum length		36.9 ± 6.0 (27-43)	45.1 ± 4.4 (40-52)	–	–	39 (36-42)	–
Tail length		24.0 ± 4.0 (18-28)	25.2 ± 2.0 (23-28)	–	–	23 (20-30)	26 (21-31)
Spicule length		–	42.6 ± 1.4 (41-45)	–	40-45	–	34 (31-36)
Ventromedian supplements		–	9 ± 0.6 (8-10)	–	–	–	8-9

**Notes:** Measurements in μm (except L, in mm), and in the form: mean ± standard deviation (range). ^a^Calculate from original description; ^b^total stylet; – this information is not available in the corresponding description.

### Description

#### Adult

Slender to very slender nematodes of medium to large size, 2.30 to 3.32 mm long. Body cylindrical, tapering toward both extremities, but more so toward anterior end. Habitus curved ventrad after fixation, especially in posterior body region. Cuticle 1.5 to 2.0 μm thick in anterior region and mid-body, and 2 to 3 μm in tail region; tylencholaimoid, consisting of a very irregularly outlined inner layer with abundant radial refractive elements, and a smooth outer layer. Lateral chord 15.5 to 24.5 μm wide or 30 to 39% of mid-body diameter. Lip region offset by deep constriction, 3.0 to 3.3 times as broad as high and one-third to two-fifths (33-41%) of body diameter at neck base; lips separated, with distinct but low, slightly protruding labial and cephalic papillae; labial disc distinct. Amphid fovea cup-shaped, its aperture 9 to 10 μm or *ca* five to ninths (51-58%) of lip region diameter. Cheilostom nearly cylindrical, lacking any differentiation. Odontostyle long and attenuated, 46 to 49 times as long as wide, 2.7 to 2.9 times as long as lip region diameter, and 1.38 to 2.05% of body length; aperture minute. Odontophore well developed, with prominent basal flanges, 0.7 to 0.8 times the odontostyle length. Guiding ring simple. Anterior region of pharynx slender and weakly muscular, basal expansion a cylindroid bulb 3.5 to 4.4 times as long as wide, 1.7 to 2.0 times as long as body diameter, and occupying 28 to 30% of total neck length; both parts of the pharynx separated by a week constriction; gland nuclei obscure in specimens examined. Nerve ring located at 115 μm (*n* = 1) from anterior end or 39% of total neck length. Cardia conoid to rounded; a ring-like structure surrounding its junction with pharyngeal base.

#### Female

Genital system didelphic-amphidelphic, with both branches equally and well developed, anterior 338 to 477 μm or 12 to 17% (*n* = 3) of body length, and posterior 323 μm or 11% (*n* = 1) of body length. Ovaries usually small, not surpassing the sphincter level, anterior 76 to 104 μm and posterior 64 to 111 μm (393 μm in only one specimen with a very large oocyte) long; oocytes arranged first in two or more rows, then in a single row. Oviduct 119 to 156 μm long or 2.3 to 2.9 times corresponding body diameter, consisting of slender part made of prismatic cells and well-developed *pars dilatata* with distinct lumen, usually containing sperm cells. Oviduct and uterus separated by a marked sphincter. Uterus tripartite, i.e. consisting of a dilated distal portion close to sphincter, a narrower intermediate section with visible lumen, and a wider proximal portion; its length 194 to 296 μm long or 3.6 to 5.9 times the corresponding body diameter; almost always containing sperm cells. Uterine eggs ovoid, 180 × 55 μm (*n* = 1), 3.3 times as long as wide. Vagina extending inwards 26 to 31 μm or 47 to 57% of corresponding body diameter, *pars proximalis* 17–23 × 14–18 μm, with sigmoid walls and surrounded by weak musculature, *pars refringens* absent, *pars distalis* well-developed 5 to 9 μm long. Vulva pre-equatorial to equatorial, transverse slit. Prerectum 17.5, 19.1 (*n* = 2) and rectum 0.8 to 1.3 anal body diameters long. Tail short and rounded; three pairs of caudal pores, two laterals, one subdorsal.

#### Male

Genital system diorchic, with opposite testes. In addition to the ad-cloacal pair, a series of 8 to 10 irregularly spaced ventromedian supplements, the posteriormost of which located outside the range of spicules. Spicules dorylaimoid, robust, weakly curved ventrad, 3.6 to 3.9 times as long as wide and 1.3 to 1.5 times as long as anal body diameter. Dorsal side regularly convex, ventral one bearing visible hump and hollow. Lateral guiding pieces 14.0 to 14.5 μm long, 7.0 to 7.4 times as long as wide. Prerectum 15.3 to 24.2 (*n* = 3), cloaca 1.4 to 1.6 the corresponding body diameters long. Tail rounded conoid, ventrally slightly concave and dorsally convex; three pairs of caudal pores, two laterals, one subdorsal.

### Diagnosis

The new population of this species is characterized by its 2.30 to 3.32 mm long body, labial disc well developed, lip region offset by constriction and 16.5 to 17.5 μm broad, odontostyle 46 to 49 μm long with minute aperture, neck 288 to 296 μm long, pharyngeal expansion 80 to 87 μm long or 28 to 30% of total neck length, uterus a tripartite tube-like structure and 194 to 296 μm long or 3.6 to 5.9 times the corresponding body diameter, *pars refringens vaginae* absent, vulva transverse (*V* = 45.4-49.7), tail short and rounded (18-28 μm in length, *c* = 94-158, *c′* = 0.6-0.9), spicules 41 to 45 μm long, and 8 to 10 irregularly spaced ventromedian supplements bearing hiatus.

### Remarks

The material herein examined fits well, morphologically and morphometrically, with the original description of *Xiphinemella esseri* by Chitwood (1957), and the subsequent re-description by De Bruin and Heyns (1991). Although some morphometrical differences were observed in the spicule length (41-45 and 40-45 μm in this study and original description by Chitwood (1957), respectively, vs 31 to 36 μm long in De Bruin and Heyns (1991). The differences found in the spicule length might be due to the fact that in our study, and also in the original description of the species, the measure of the arc of the spicule are provided, whereas in the De Bruin and Heyns’ description most likely (it is not specifically mentioned in the description) the measure of the chord of the spicule was taken.

### Molecular characterization and phylogenetic position of *Xiphinemella esseri*

**Figure 2:**
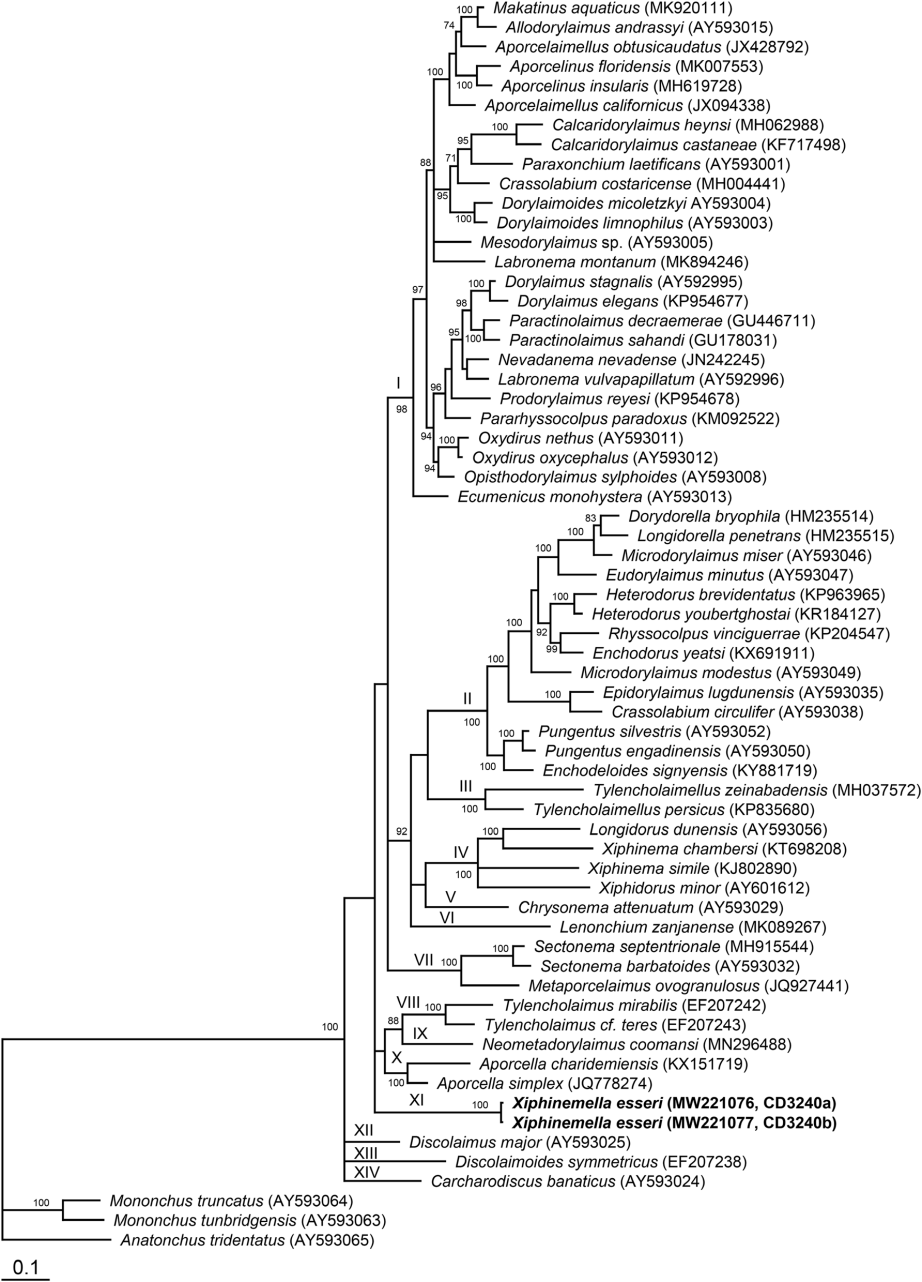
Bayesian 50% majority rule consensus tree as inferred from the D2-D3 expansion segments of 28S rRNA gene sequence alignment under the GTR + I + G model. Posterior probability values more than 70% are given on appropriate clades. New sequences are indicated by bold letters.

The D2-D3 of 28S rRNA gene alignment generated with modified parameters (gap opening = 5 and gap extension = 3) was 907 bp in length and contained 64 sequences. Phylogenetic relationships of *X. esseri* within selected dorylaimid nematodes are given in Figure 2. Two sequences obtained from two specimens of *X. esseri* differed in 3 bp and clustered together (Clade XI). Relationships of *X. esseri* with other dorylaims were not resolved. This result is most likely due to the lack of 28S rDNA sequences of other leptonchid genera.

**Figure 3:**
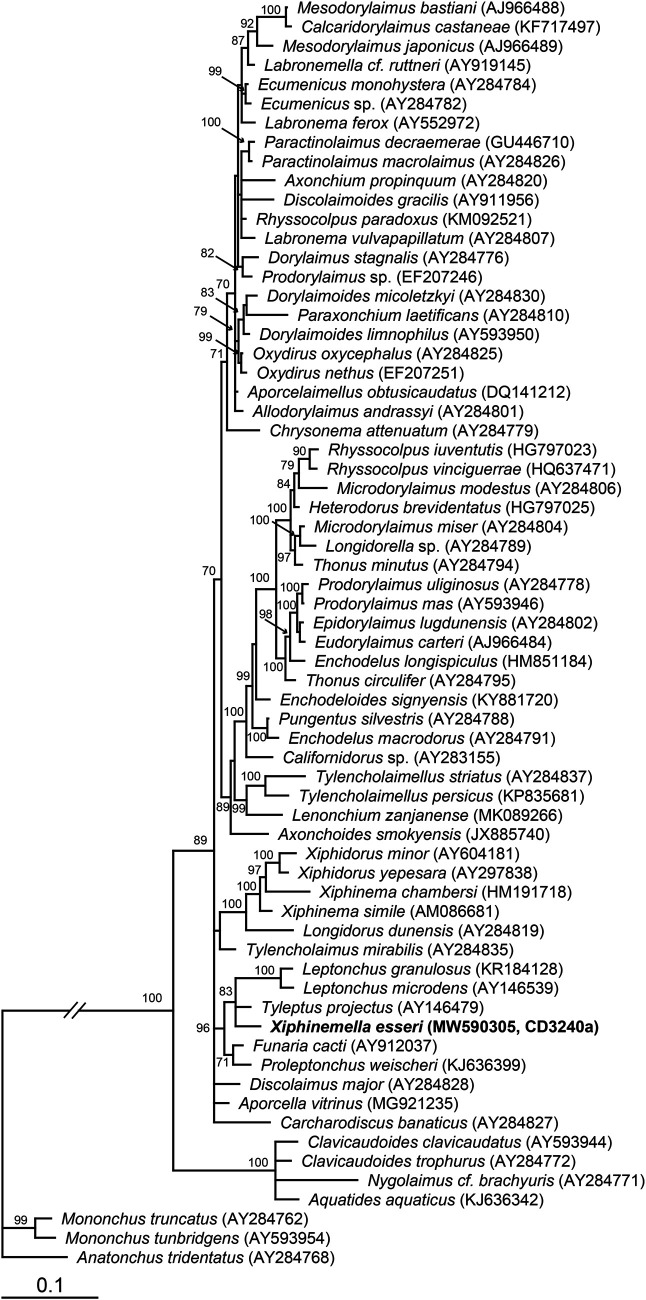
Bayesian 50% majority rule consensus tree as inferred from the 18S rRNA gene sequence alignment under the GTR + I + G model. Posterior probability values more than 70% are given on appropriate clades. A new sequence is indicated by bold letters.

The 18S rRNA gene alignment was 1754 bp in length and contained 66 sequences. Phylogenetic relationships of *X. esseri* within selected dorylaimid nematodes are given in Figure 3. Sequences obtained from *X. esseri* clustered with those of several other genera of Leptonchidae (*Leptonchus, Tyleptus, Funaria*, and *Proleptonchus*) in a well-supported clade (posterior probabilities BI: 96). This result supports and justifies the classification of *Xiphinemella* under the family Leptonchidae.

### Distribution and biological considerations

*Xiphinemella* species have been reported associated in many geographical regions with forest trees and herbaceous plants such as cowpea (*Vigna unguiculata* (L.) Walp.), rice (*Oryza sativa* L.) and others (De Bruin and Heyns, 1991). However, there are no reports on the feeding habits of these species. So far, the species *X. esseri* has been found only in Florida, where it is often associated with oak trees such as live oak and Spanish oak or Southern red oak (*Quercus falcata* Michx.). The original population described by Chitwood was collected from a Spanish oak growing in the north west section of the city of Gainesville, an area now developed into residential subdivisions and apartment complexes. For their re-description, De Bruin and Heyns (1991) used specimens collected by R. P. Esser from weeds associated with *Magnolia grandiflora* L. in Brandon (about 200 miles south of Gainesville). The specimens used for this study were collected from live oaks in a tree farm operation located in the city of Alachua (latitude 29°88′00.63″ N, longitude 82°48′26.41″ W). The nematode records of the Florida Department of Agriculture and Consumer Services indicate that movement of this nematode with the trade of infested oak trees to other localities in and outside Florida has occurred for a long time. Many longidorids such as *Longidorus longicaudatus* (Siddiqi, 1962), species of *Xiphinema americanum* group and *Xiphinema setariae* (Luc, 1958) feed on oak roots and are associated with *X. esseri*. It seems *X. esseri* may also have plant parasitic habits as suggested by Chitwood (1957), although the parasitism of this species has not been scientifically demonstrated. For the convenience of taxonomists, we have deposited nine females and seven males mounted on glass slides in the nematode collection of the National Museum of Natural Sciences, Madrid, Spain; in addition, three females, two males and five juveniles were sent to the United States Department of Agriculture Nematode Collection, Beltsville, MD, USA.
